# Multi-recurrent Asynchronous Bilateral Malignant Phyllodes

**DOI:** 10.7759/cureus.57936

**Published:** 2024-04-09

**Authors:** Saeeda Yasmin, Nida Rasul, Muhammad Ali Hassan, Ahmed Ehsan Rabbani, Arham Yahya Rizwan Khan

**Affiliations:** 1 General Surgery, Shifa International Hospital Islamabad, Islamabad, PAK; 2 Medicine, Shifa College of Medicine, Shifa Tameer-e-Millat University, Islamabad, PAK; 3 Community Medicine, HBS Medical and Dental College, Islamabad, PAK

**Keywords:** phyllodes tumor, malignant phyllodes tumor, multi-recurrent, bilateral phyllodes tumor, asynchronous

## Abstract

In this comprehensive study, we present an exceptionally rare case characterized by the occurrence of multi-recurrent asynchronous bilateral malignant phyllodes tumors. Phyllodes tumors, known for their rapid growth, originate within the stromal tissue of the breast and predominantly manifest as benign entities. Our case stands out as an extraordinary anomaly, not only due to its bilateral malignant nature but also owing to the manifestation of a multi-recurrent pattern on both sides. This unprecedented presentation underscores the complexity and heterogeneity of malignant phyllodes tumors, necessitating further in-depth investigation to unravel the underlying mechanisms driving their aggressive behavior and to explore innovative therapeutic strategies aimed at optimizing patient outcomes and prognosis.

## Introduction

Phyllodes tumors resemble fibroadenomas and express an overgrowth of the fibrous component. They are divided into benign, malignant, and borderline categories based on their histological characteristics.

There is an inherent tendency for recurrence and/or metastatic potential, which is determined by the histological grade of the tumor. Even though diagnosis is mainly on a histopathological basis, differentiating between a phyllodes tumor and a fibroadenoma may be difficult on a core biopsy specimen [1]. Features, such as stromal hypercellularity, and immunohistochemistry markers, such as Ki67, topoisomerase II alpha, and CD34, along with clinical suspicion, can lead to a diagnosis on a core biopsy [2].

They are very rare tumors, accounting for 0.3-1% of breast tumors [3]. Thirty-five to 55 seems to be the age group most involved, and only a few cases have been reported in men [4]. The National Comprehensive Cancer Network guidelines for breast cancer suggest that for phyllodes tumors greater than 3 cm, surgical excision with 1 cm clear margins without axillary staging is the primary treatment, regardless of whether the tumor is benign, borderline, or malignant [5]. It has been observed that younger age, larger tumor size, higher tumor grade, and positive margins might be associated with lower rates of local recurrence-free survival [6]. Fibroproliferation within the surrounding breast tissue and necrosis are also associated with higher local recurrence rates [7].

We present a rare case of multi-recurrent asynchronous bilateral malignant phyllodes tumors. The case is of a 36-year-old woman who presented with primary malignant phyllodes tumors, and following bilateral mastectomies, she developed multiple recurrences. After receiving eight cycles of radiotherapy, she developed metastasis to the ribs and lungs and has since been lost to follow-up.

## Case presentation

A 36-year-old woman presented to the Shifa Foundation with a right breast lump in May 2012. Before her presentation to the Shifa Foundation, all of her previous treatments were done at a public-sector hospital. The first time she detected a lump in her right breast was during her pregnancy in 2006. She was treated with the excision of a giant fibroadenoma. She presented at the public hospital again in January 2008 with a one-year history of a right breast lump that was gradually increasing in size. A core biopsy revealed a malignant phyllodes tumor. A right simple mastectomy with clear margins was carried out in February 2008, and histopathology confirmed malignant phyllodes T3NxM0. She was advised to undergo radiotherapy, but she refused.

**Figure 1:**
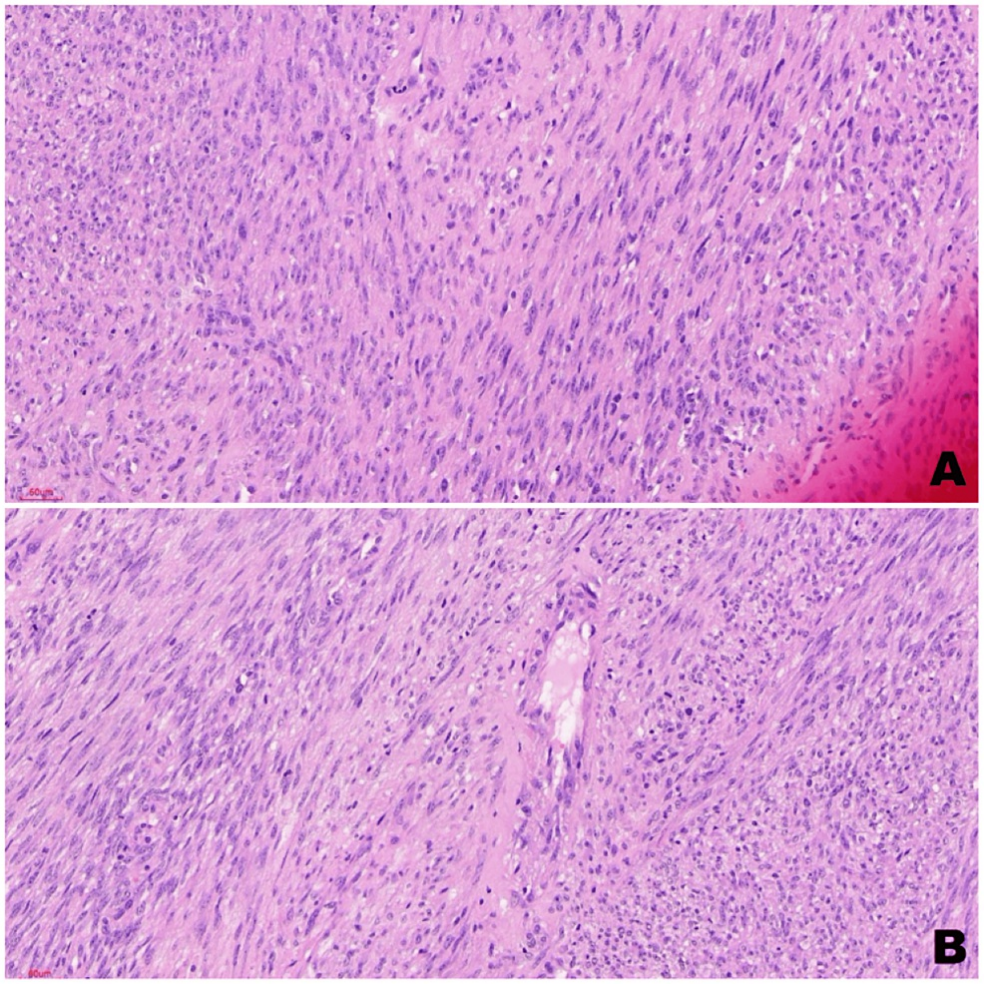
(A) Stroma shows spindle-shaped cells, hypercellularity, and scattered mitotic figures. (B) Compressed ducts lined by a double layer of epithelial and myoepithelial cells

In May 2009, the patient presented to a public hospital with left-sided malignant phyllodes. Left modified radical mastectomy (MRM) and axillary lymph node dissection were performed. Following surgery, histopathology revealed a tumor size of 19x11x9 cm with clear margins and mitosis of 11/10 HPF. Densely packed stromal cells showed mild to moderate anaplasia, with stromal tissue showing more than ductal overgrowth (Figure 1). Twenty-nine lymph nodes were recovered and were found to be tumor-free. CT of the chest, abdomen, and pelvis showed no evidence of metastasis. Local chest wall radiotherapy was advised, but the patient refused.

**Figure 2:**
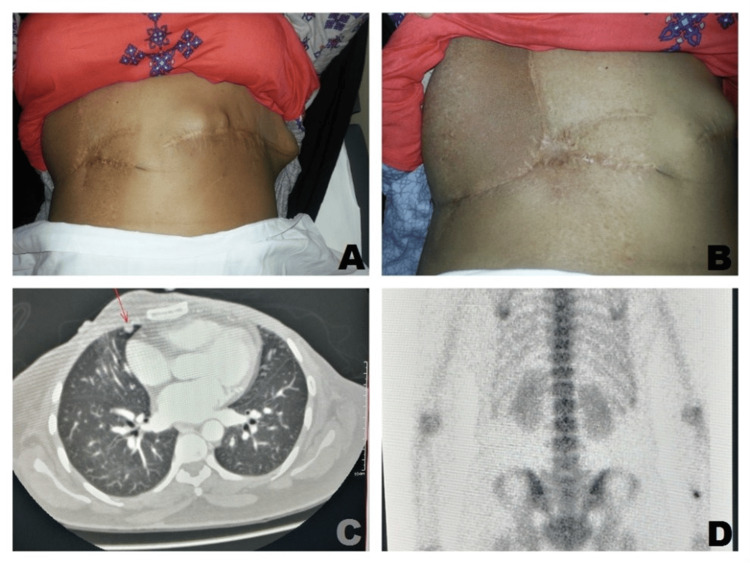
(A) Bilateral mastectomy scars showing new lumps on the left side. (B) Latissimus dorsi flap reconstruction in the right axilla. (C) CT showing metastasis to the lung. (D) Bone scan with rib metastasis

In May 2012, the patient presented to the Shifa Foundation with multiple lumps bilaterally in the chest wall and also in the right axilla. On examination, there were bilateral chest wall lumps and a fungating lesion with pus and blood-stained discharge from axillary lumps. On USG, the patient was also 12 weeks pregnant. On MRI, multiple lobulated, intensely enhancing, predominantly low T1 and high T2 soft tissue masses in the chest wall were reported. Termination of pregnancy on medical grounds was carried out with the patient's consent. A bilateral, wide local excision of chest wall tumors was performed with clear margins. Reconstruction was done in the right axilla with a latissimus dorsi flap (Figure 2B). Bilateral adjuvant radiotherapy was administered to the patient. A left chest wall biopsy revealed recurrent malignant phyllodes with clear margins and mitosis of 2/10, while a right-sided biopsy showed a malignant phyllodes tumor of 28x10x8 cm with clear margins and heterogeneous stromal hypercellularity. Mitosis was 10/10 HPF. Sixteen lymph nodes were retrieved and were tumor-free.

The patient presented with a second recurrence on the left side in October 2012. Two solid lesions were found along the mastectomy scar (Figure 2A). These lesions subsided with left chest wall radiotherapy.

In February 2014, the patient had a third recurrence on the left side. Surgical excision was done with clear margins. This time, the patient refused adjuvant radiotherapy due to financial constraints.

The patient presented once more in July 2015, this time with a fungating growth at the medial end of the left side of the chest scar. A CT scan showed a large lobulated fungating peripherally enhancing mass with central necrosis invading the left anterior chest wall, measuring approximately 12x10x7.2 cm. A wide local excision was done with clear margins, followed by radiotherapy.

The patient came back with yet another recurrence in November 2015. A wide local excision of the nodule was carried out. She was advised to undergo radiotherapy but was not compliant with it. In January 2016, the patient once again presented with the appearance of a left-sided chest wall nodule. A wide local excision was performed, showing malignant phyllodes. Metastatic workup showed lung and rib metastasis (Figure 2C, 2D). A medical oncologist advised chemotherapy. Ten cycles of chemotherapy were given until January 2017, with a reduction in the size of chest nodules and metastatic lesions. The patient had a left-sided chest wall recurrence with fungation again and was resistant to chemotherapy. As she was a surgically irresectable patient, she was referred to an oncologist for palliative chemoradiation for symptomatic relief. Later, the patient was lost to follow-up.

## Discussion

The phyllodes tumor arises in the stroma of the breast and is a rare fibroepithelial neoplasm. Approximately 0.3-0.9% of all breast cancers fall into the category of these fibroepithelial neoplasms. The most common age group for the presentation of these tumors is females between 40 and 60 years of age [8]. Phyllodes tumors are treated as aggressive breast tumors as they usually grow rapidly, and although they are uncommon, they have the potential for malignant transformation. The risk of malignant transformation is approximately 2.1 per one million women [8].

Phyllodes tumors are often misdiagnosed as fibroadenomas due to their structural similarities. In our patient, a core biopsy confirmed a malignant phyllodes tumor. The phyllodes tumor expresses a distinct leaf-like structure on histology. Core needle biopsy highlights four distinguishing features of phyllodes tumors: increased stromal cellularity, stromal overgrowth, fragmentation, and adipose tissue within the stroma.

A review of the literature showed multiple studies about possible bad prognostic factors for local recurrence and distant metastasis. Kapiris et al. reported tumor size and negative resection margin as the main factors for local recurrence, while Asoglu et al. found stromal overgrowth as the sole important indicator of distant metastasis [9,10]. Onkendi et al. reported large tumor size, high mitotic count, stromal overgrowth, and high cellularity as indicators of high local recurrence and less disease-free survival [11]. Our patient had a very large tumor size at the time of presentation with marked stromal overgrowth, a high mitotic count, and high nuclear pleomorphism, all indicative of a high-grade malignant phyllodes tumor with a high potential for recurrence.

The mainstay of treatment for phyllodes tumors is surgical resection. Smaller tumors are often treated by wide local excision with clear margins, while mastectomy is more suitable for larger tumors. In a study conducted on the comparison of wide local excision and mastectomy to decrease the chances of recurrence, mastectomy remains the treatment of choice [10]. In our patient, she was first treated with a simple right mastectomy, followed by a bilateral modified radical mastectomy, and axillary lymph node clearance after bilateral recurrence at different timings. She also received multiple cycles of chest wall radiotherapy. A meta-analysis published in 2019 suggested the use of radiotherapy regardless of the type of surgery to decrease the rate of local recurrence and distant metastasis [12]. Though our patient had aggressive surgeries with favorable resection margins and chest wall radiotherapy, multiple recurrences still occurred. It seems as if radiotherapy and surgery were worthless to our patient, and we don't know the possible cause. Shpitz et al. suggested that recurrent phyllodes tumors seemed to show progression to more malignant phenotypes with high proliferative indices and high p53 expression [13]. It may be hypothesized that the cytogenetic aspects of malignant phyllodes in our case may have contributed to the particularly aggressive behavior. Hematogenous metastasis to the lungs remains the main form of distant metastasis in malignant phyllodes tumors. Our patient first presented in 2008; however, metastasis to the lungs and ribs occurred in 2016. The average time for distant metastasis to present has been reported to be 25.6 months, while in our patient, it occurred after eight years, after multiple local recurrences [9].

This case is unique because, despite aggressive surgeries, radiotherapy, and regular follow-up for an eight-year period, repeated local recurrences could not be prevented, and distant metastasis ultimately occurred. While the literature has reported cases of aggressive unilateral as well as bilateral malignant phyllodes, to our knowledge, a case of multi-recurrent asynchronous malignant phyllodes with such an aggressive presentation is the first to be reported [14,15].

## Conclusions

The unprecedented presentation of this case underscores the complexity and heterogeneity of malignant phyllodes tumors. Further research is warranted on this subject to find the exact cause of the aggressive attitude of such tumors. Additionally, there is a pressing need to explore alternative therapeutic approaches that can be employed in the management of such patients, aiming to optimize treatment outcomes and prognosis.
